# Molecular Actions of Ovarian Cancer G Protein-Coupled Receptor 1 Caused by Extracellular Acidification in Bone

**DOI:** 10.3390/ijms151222365

**Published:** 2014-12-03

**Authors:** Feng-Lai Yuan, Ming-Dong Zhao, Li-Bo Jiang, Hui-Ren Wang, Lu Cao, Xiao-Gang Zhou, Xi-Lei Li, Jian Dong

**Affiliations:** 1 Department of Orthopedic Surgery, Zhongshan Hospital, Fudan University, Shanghai 200032, China; E-Mails: bjjq88@163.com (F.-L.Y.); zhaomingdong@medmail.com.cn (M.-D.Z.); j198611@163.com (L.-B.J.); 13788999017@163.com (H.-R.W.); caolu922@126.com (L.C.); zhou.xiaogang@zs-hospital.sh.cn (X.-G.Z.); li.xilei2@gmail.com (X.-L.L.); 2 Department of Orthopaedics and Central Laboratory, the Third Hospital Affiliated of Nantong University, Wuxi 214041, China

**Keywords:** extracellular acidification, OGR1, osteoclasts, osteoblasts, endplate chondrocytes

## Abstract

Extracellular acidification occurs under physiologic and pathologic conditions, such as exercise, ischemia, and inflammation. It has been shown that acidosis has various adverse effects on bone. In recent years there has been increasing evidence which indicates that ovarian cancer G protein-coupled receptor 1 (OGR1) is a pH-sensing receptor and mediates a variety of extracellular acidification-induced actions on bone cells and other cell types. Recent studies have shown that OGR1 is involved in the regulation of osteoclast differentiation, survival, and function, as well as osteoblast differentiation and bone formation. Moreover, OGR1 also regulates acid-induced apoptosis of endplate chondrocytes in intervertebral discs. These observations demonstrate the importance of OGR1 in skeletal development and metabolism. Here, we provide an overview of OGR1 regulation ofosteoclasts, osteoblasts, and chondrocytes, and the molecular actions of OGR1 induced by extracellular acidification in the maintenance of bone health.

## 1. Introduction

Bone and cartilage are the two primary components that form the skeleton in vertebrates [1]. These two tissues consist of three specific cell types scattered within the extracellular matrix (ECM): Bone-forming osteoblasts, bone-resorbing osteoclasts in bone, and cartilage-forming chondrocytes in cartilage [2]. The macroscopic and microscopic structural changes in bone are influenced by physiologic and pathologic conditions, such as mechanical stress, hypoxia, and acidosis [2,3,4,5]. Because of fractures, hypoxia, inflammation, and tumors, the bone microenvironment has long been known to be acidic [6,7]. Acidic pH can also result from hormonal, growth factor, or cytokine stimulation of bone cell metabolism [8]. It has been shown that parathyroid hormone and insulin-like growth factor-1 (IGF-1) lead to a rapid acid efflux from osteoblasts [9]. The cartilage exists in an extracellular environment where the pH of the interstitial fluid is much more acidic than most other tissues. The avascular nature of cartilage causes hypoxia within the ECM which may lead to acidosis in the cartilage microenvironment [10].

Although the sensing mechanism of extracellular acid remains largely unknown, great breakthroughs have also been made toward understanding the cellular sensory mechanisms by which cells detect changes in the extracellular pH in such a sensitive manner. It has been shown that transient receptor potential V1 (TRPV1) is a calcium-permeable channel which is modulated or activated by extracellular protons [11]. Another family of molecular acid sensors is the acid-sensing ion channels (ASICs), which encode at least six different ASIC subunits, including ASIC1a, ASIC1b, ASIC2a, ASIC2b, ASIC3, and ASIC4 [12]. Proton-sensing G protein-coupled receptors (GPCRs) are emerging as a new class of acid sensors on a wide range of cell types that transduce signals through heterotrimeric G proteins [13]. The family of GPCRs is involved in cancer cell proliferation, apoptosis, metastasis, angiogenesis, osteoclast differentiation and survival, dendritic cell (DC) activities, alteration of DC functions, and insulin secretion; some of these GPCRs have turned out to be sensors for extracellular acidosis [14]. The transcripts of proton-sensing GPCRs, particularly ovarian cancer G protein-coupled receptor 1 (OGR1), are widely distributed and expressed on bone cells that are involved in the regulation of osteoclast differentiation, survival, and function, osteoblast differentiation and bone formation, as well as apoptosis of endplate chondrocytes in intervertebral discs [13,15,16]. This review will summarize our current knowledge regarding OGR1 in bone, and will highlight recent advances in bone metabolism. It has been showed that in bone cells metabolic acidosis increased [Ca^2+^]i from intracellular stores through activation of OGR1.

## 2. Proton-Sensing GPCRs

**Figure 1:**
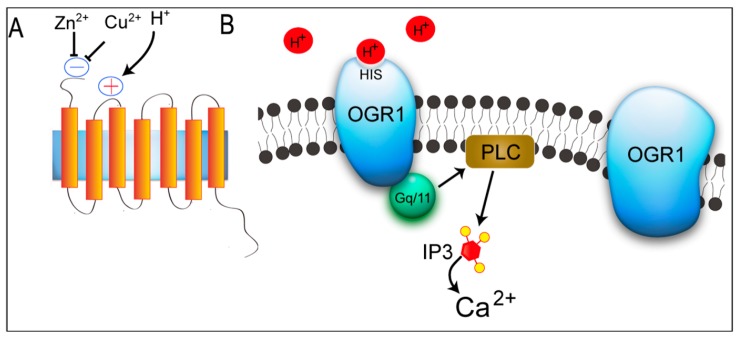
Activation of ovarian cancer G protein-coupled receptor 1 (OGR1) by extracellular acidification. (**A**) Proton has been suggested as an agonists of OGR1. Cu^2+^ and Zn^2+^ inhibit pH-dependent OGR1 activation; and (**B**) His residues that have been reported to be involved in proton-sensing process are bolded and underlined in OGR1. The interaction between protons and His residues of OGR1 leads to the activation of Gq/11/PLC/Ca^2+^ pathway. PLC: phospholipase C; Gq/11: Gq/11 protein; IP3: inositol 1,4,5-trisphosphate.

There are four members in the GPCR family (OGR1, G protein-coupled receptor 4 (GPR4), T cell death-associated gene 8 (TDAG8), and G2 accumulation protein (G2A)), which have previously been identified as receptors for lysolipids (sphingosylphosphorylcholine (SPC), lysophosphatidylcholine (LPC), and psychosine (galactosylsphingosine)) [17,18,19,20]. Recent studies, however, have shown that these GPCRs also sense extracellular protons through histidine residues of receptors and are coupled to G-proteins to stimulate intracellular signaling pathways. Ludwig *et al.* [17] first reported that OGR1 and GPR4 are proton-sensing receptors and coupled to Gq/11 and Gs proteins by regulating activation of the phospholipase C (PLC)/Ca^2+^ and adenylyl cyclase/Cyclic Adenosine monophosphate (cAMP) signaling pathways, respectively. OGR1 is inactive at pH 7.8, but fully activates inositol phosphate (IP) formation at pH 6.8. Moreover, Ludwig *et al.* [17] showed that GPR4 senses extracellular protons, but GPR4 activates the Gs-adenylyl cyclase-cAMP signaling pathway; however, they were not able to find any effect of SPC and LPC, which were previously reported to activate OGR1 and GPR4. In 2004, Murakami *et al.* [21] reported that G2A functions as a proton-sensing GPCR, like OGR1 and GPR4, by regulating multiple classes of G-proteins, including G13 and Gi/Go in signaling. Wang *et al*. [22] reported that TDAG8 senses extracellular protons and have recently been identified as proton-sensing or extracellular pH-responsive GPCRs, leading to activation of the cAMP signaling pathway. Among these receptors, OGR1is widely distributed and expressed on bone cells that take part in bone metabolism. OGR1, also named as GPR68 (G protein-coupled receptor 68), is a 365 amino acid multi-pass membrane protein that is expressed in testis, spleen, bone, lung, brain and placenta [17,23]. OGR1 selectively binds both protons and bioactive lipids and acts through Gi and Gq proteins-mediated processes [24,25]. Under the acidic conditions, pH-sensing activity of OGR1depends on several His residues that reside in the extracellular domains of this seven-pass transmembrane protein, resulting in the activation of intracellular signaling pathways (Figure 1).

## 3. OGR1 and Bone

### 3.1. Causes of Acidosis

Tissue acidosis can result from a number of systemic and local causes. Systemic acidosis, which is caused by pathologic conditions, such as renal and respiratory disease, anemias, and diabetes, leads to abnormal cell function throughout the body [26]. Systemic acidosis can also be caused by high levels of protein intake (or acid feeding), aging, or the menopause [27]. Localized extracellular acidosis occurs due to ischemia and hypoxia caused by diabetes, wounds, inflammation, infection, and tumors [6]. Extracellular acidosis can also result from hormonal, growth factor, or cytokine stimulation of cell metabolism [8]. The deleterious effect of acidosis on the bone has long been known. In particular, proton-sensing GPCRs, such as OGR1, respond to low pH and play a critical role in the regulation of osteoclast function, osteoblast differentiation, and apoptosis of endplate chondrocytes in intervertebral discs [8,13,16,27,28].

### 3.2. OGR1 and Osteoclasts

In 2005, Komarova *et al.* [29] first showed that OGR1 is expressed in osteoclast-like cells differentiated *in vitro* from RAW 264.7 cells induced by receptor activator of NF-κB ligand (RANKL). In addition, RANKL increases the levels of expression of OGR1 mRNA in RAW 264.7 pre-osteoclast-like cells. Pereverzev *et al.* [28] also showed that reduction of extracellular pH in osteoclasts resulted in nuclear translocation of NFATc1, a downstream mediator of RANKL differentiation effects, although no specific physiologic role for OGR1 in that process was demonstrated. Localization of OGR1 in the plasma membrane area suggests that OGR1 may act as a functional receptor on these cells. Consistent with the differences in transcript levels, the immunofluorescence staining of OGR1 in differentiated osteoclast-like cells is more intense than undifferentiated RAW 264.7 cells. Yang *et al.* [16] reported that OGR1 is also significantly up-regulated in tibias and femurs after 2 days of colony stimulating factor-1 (CSF-1) injections based on the ability of CSF-1 to restore osteoclast populations in the CSF-1-null toothless (*csf1tl*/*csf1tl*) osteoporotic rat. The expression of OGR1 mRNA and protein was also observed by microarray, real-time RT-PCR, and immunoblotting when mouse bone marrow mononuclear cells (BMMs) were treated with RANKL to induce osteoclast differentiation. Specific inhibition of OGR1 by anti-OGR1 antibody and OGR1-specific RNA interference (RNAi) suppressed RANKL-induced differentiation of both BMMs and RAW 264.7 cells *in vitro*. It has been proposed that OGR1 is expressed during osteoclastogenesis *in vivo* and *in vitro* and is crucial for osteoclast differentiation; however, the molecular mechanism of OGR1 in regulating osteoclast differentiation and function remains unclear.

Pereverzev *et al*. [28] recently detected extracellular acidification enhances osteoclast survival. This study directly supports the potential activation of OGR1 in mediating osteoclast survival during extracellular acidification. Ca^2+^ signaling in osteoclasts is crucial for cellular functions, including motility, differentiation, and bone-resorbing activity [16,28,30]. Recent findings suggest that OGR1 is essential for the extracellular acidification-induced increase in [Ca^2+^]i levels in osteoclasts [28]. More interestingly, OGR1-mediated calcium signaling occurs in osteoclasts during extracellular acidosis, which contributes to acidosis-induced osteoclast survival. OGR1 activation in osteoclast enhances survival by inducing the activation of protein kinase C (PKC) that may affect the phosphorylation status of pro- or anti-apoptotic proteins, or stimulate extracellular signal-regulated kinases 1 and 2 (ERK1/2) signaling, which is critical for osteoclast survival [28]. Similar observations were reported in a more recent study in OGR1 deficient mice which were generated by homologous recombination [31]. OGR1 deficiency led to a decrease in osteoclast numbers, suggesting that OGR1 may play an important role in osteoclastogenesis. A pH-dependent survival effect of osteoclasts was also detected. However, overall abnormality in the bones of OGR1 deficient mice was not observed. It is possible that the defect in osteoclast numbers and/or their response to pH changes will affect some biological functions under certain pathological conditions.

### 3.3. OGR1 and Osteoblasts

Ludwig *et al.* [17] reported that the expression of OGR1 protein is detected in active osteoblasts, lining cells on the bone surface, and matrix-embedded osteocytes by immunohistochemistry. Moreover, Tomura *et al.* [32] reported OGR1 is predominantly expressed in human osteoblastic cells (NHOst). Several groups have investigated how acidosis works via OGR1 in osteoblasts [15,17,32,33]. Acidosis activates OGR1 to elevate [Ca^2+^]i levels via Gq stimulation, inducing cyclooxygenase 2 (COX-2) mRNA and protein expression in human osteoblastic cells. This leads to the production of prostaglandin E_2_ (PGE_2_), which is reported to activate osteoblasts to RANKL expression, a key cytokine involved in osteoclast differentiation [32]. Moreover, knocking down OGR1 with siRNA inhibits acidosis-induced COX-2 expression in a human osteoblastic cell line [32]. Tomura *et al.* [32] used YM-254890, a Gq antagonist that specifically inhibits Gq activation, and PLC inhibitors that significantly inhibit acid-induced COX-2 expression and subsequent PGE_2_ production, suggesting that the OGR1/Gq/11/PLC pathway is involved in COX-2 expression and PGE_2_ production in osteoblasts. This cascade from OGR1 to COX-2 and RANKL in osteoblasts might be an event in the induction process by acidic circumstances.

Frick *et al.* [15] previously reported that acidosis also leads to an increase in net Ca^2+^ efflux from bone. Recent studies have demonstrated that the OGR1 antagonist, Cu^2+^, significantly decreases acid-induced bone net Ca^2+^ efflux, a marker of bone resorption, in cultured neonatal mouse calvariae [33]. To further support OGR1 as a prime candidate as an osteoblastic H^+^ sensor, Frick *et al*. [33] perfused Chinese hamster ovary (CHO) cells transfected with mouse OGR1 cDNA. Eventually, a rapid increase in the intracellular calcium ([Ca^2+^]i) levels were also detected in OGR1-transfected CHO cells in response to an acidic medium, which acts as a second messenger to mediate the effects of acidosis on osteoblasts and results in increased osteoclastic bone resorption by inducing increased COX-2 and RANKL expression.

### 3.4. OGR1 and Chondrocytes

In 2003, Ludwig *et al.* [17] first reported that the expression of OGR1 is also specifically expressed in chondrocytes of hypertrophic cartilage. Experiments in our laboratory using rat lumbar endplate chondrocytes have been shown that high levels of OGR1 mRNA and low levels of G2A and TDAG8 mRNA in rat endplate chondrocytes were detected by RT-PCR analysis [13]. Interesting results were noted when cultures of rat lumbar endplate chondrocytes were exposed to acidosis; the mRNA levels of OGR1 increased in response to acidosis, whereas the mRNA levels of the other receptors were unchanged. Our data suggest that OGR1 is responsive to pH in endplate chondrocytes [13].

**Figure 2:**
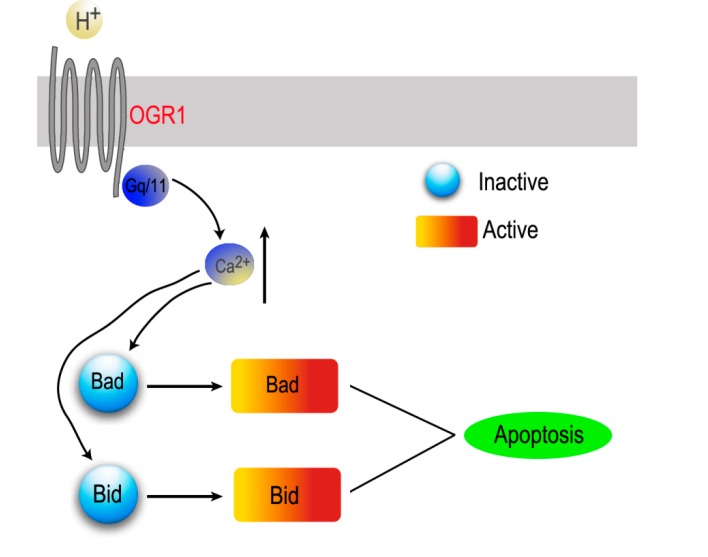
Schematic diagram of the potential mechanism of proton-sensing receptor OGR1 involved in acid-induced apoptosis of endplate chondrocytes.

Additionally, we demonstrated that OGR1 is involved in apoptosis of endplate chondrocytes induced by extracellular acid in the rat intervertebral disc. Acid-induced [Ca^2+^]i increase via OGR1 is responsible for endplate chondrocytes apoptosis. A mechanism for OGR1 in endplate chondrocytes in an acidic environment has been proposed wherein cell apoptosis is related to OGR1-mediated apoptosis via down-regulation of calcium-activated signaling pathways, such as Bid and Bad, and inhibition of caspase-3 and poly(ADP-ribose) polymerase (PARP) activity (Figure 2).

## 4. Conclusions

In the present review, we have discussed extracellular acidification regulating a wide range of cellular functions and their mechanisms, especially focusing on proton-sensing OGR1 in bone cells. Increasing evidence supports a central role for the OGR1 in bone biology and disease; however, there were several limitations to the study that will require further exploration. The role of OGR1 in bone biology and disease should be confirmed in several animal models. It will also be important to determine whether or not there are any developmental changes during bone disease in OGR1-deficient mice. Interestingly, by observing the development of knockout (KO) mice, Li *et al.*. [31] has elucidated essential roles for OGR1 in regulating osteoclastogenesis. *TDAG8* gene mutation in ovariectomized miceresulted in an increase in osteoclastic activity, suggesting an inhibitory role of TDAG8 in osteoclastic bone resorption in osteoporosis [34]. Further investigation into the regulatory mechanisms of OGR1 is necessary for developing effective therapeutic strategies for the treatment of bone diseases.
